# Identification and Functional Analysis of Cystathionine Beta-Synthase Gene Mutations in Chinese Families with Classical Homocystinuria

**DOI:** 10.3390/biomedicines13040919

**Published:** 2025-04-09

**Authors:** Xin Liu, Xinhua Liu, Jinfeng Liu, Junhong Guo, Danyao Nie, Jiantao Wang

**Affiliations:** Shenzhen Eye Hospital, Shenzhen Eye Medical Center, Southern Medical University, Shenzhen 518040, China; ljy_szeh@126.com (X.L.); gjh0759@163.com (J.G.)

**Keywords:** homocystinuria, exome, pathomechanism, CBS, minigene, RNA splicing

## Abstract

**Background:** Homocystinuria caused by cystathionine β-synthase (CBS) deficiency is the most common congenital disorder related to sulfur amino acid metabolism, manifested by neurological, vascular, and connective tissue involvement. **Methods:** This study analyzed the pathogenic gene and molecular mechanism of two classic homocystinuria families through whole exome sequencing and in vitro experiments including minigene assay and expression analysis. **Results:** Both probands presented with ectopia lentis, high myopia, and abnormally elevated homocysteine level, but one of them had more severe clinical manifestations, including general growth retardation, mild intellectual disability, and severe pectus excavatum. Their family members were phenotypically normal but presented slightly higher levels of homocysteine in plasma. Whole exome sequencing revealed that the two probands carried c.833T>C (p.Ile278Thr) and c.1359-1G>C, and c.919G>A (p.Gly307Ser) and c.131delT (p.Tle44Thrfs*38) compound heterozygous mutations in the *CBS* gene, respectively. Bioinformatics and in vitro functional analysis showed that the c.1359-1G>C mutation affects the normal splicing of *CBS* gene, resulting in the production of two abnormal transcripts and the production of two truncated proteins. One of the c.1359-1G>C splicing events (c.1359_1467del) and c.131delT (p.Tle44Thrfs*38) both lead to a significant decrease in CBS mRNA and protein levels. **Conclusions:** Accurate diagnosis of patients with homocystinuria is of great importance for timely and effective treatment, as well as for the provision of appropriate genetic counseling and prenatal diagnosis guidance to the affected families.

## 1. Introduction

Classical homocystinuria (MIM# 236200), also known as homocysteinemia type 1, is a rare congenital metabolic disorder caused by deficiency of cystathionine β-synthase (CBS), an enzyme that catalyzes the transsulfuration of homocysteine (Hcy) to cystathionine with the help of its cofactor pyridoxine [1]. Impaired CBS activity leads to the elevation of both the methionine and Hcy levels in the blood and urine, which are the main indicators for diagnosing this disease [2]. In addition to CBS deficiency, elevated plasma Hcy can also originate from (1) genetic defects in Hcy metabolism (e.g., methylenetetrahydrofolate reductase (*MTHFR*) mutations impairing remethylation), (2) acquired deficiencies of vitamin cofactors (B6, B12, or folate) required for Hcy processing, or (3) secondary metabolic disturbances associated with aging or chronic kidney disease [3,4]. Therefore, integrating genetic testing with multidisciplinary clinical assessments is critical to delineate the pathogenic mechanisms of hyperhomocysteinemia and implement targeted interventions. The classical homocystinuria patients are usually normal at birth and may suffer from stunting in infancy. Classical homocystinuria leads to elevated levels of homocysteine and methionine in blood and urine, and decreased levels of cystathionine and cysteine [2]. Patients who did not receive treatment during infancy may develop a variety of diseases, including ocular anomalies (severe myopia and ectopia lentis), disorder of central nervous system (mental defects and convulsions), premature arteriosclerosis, and thrombosis, skeletal deformities (osteoporosis and scoliosis), and other manifestations [5,6]. To date, there is no effective cure for homocystinuria, and the standard treatment focuses on reducing Hcy levels by supplementing the patient with betaine, folic acid, and pyridoxine (vitamin B6) to make them as close to the normal range as possible. Special diet and medicines treatment in the very early stages of the disease can effectively reduce blood Hcy levels and prevent the development of complications [7].

The worldwide incidence rate of homocystinuria is approximately 1/344,000, but with considerable variations between individual countries [8,9]. The *CBS* gene is located on the long arm of chromosome 21 and encodes a homotetrameric protein, with each subunit consisting of three structural domains. At present, more than 200 *CBS* mutations have been reported to be causally associated with classical homocystinuria (HGMD Professional^®^ 2024.1 total; The Human Gene Mutation Database, 2019). Of these, more than 80% are missense mutations, with p.Gly307Ser and p.Ile278Thr being the two most commonly reported mutations around the world [10,11].

In this study, the clinical and genetic features of two families with classical homocystinuria from southern China were investigated. Whole exome sequencing identified candidate disease-causing compound heterozygous mutations, which were from the parents of the proband, respectively. The pathogenicity of the new splicing mutation was verified by using the minigene assay and in vitro functional analysis. These results may help to clarify the molecular pathogenesis and clinical diagnosis of CBS-related homocystinuria, providing evidence for the potential development of effective genetic therapies to treat this disease in the future.

## 2. Materials and Methods

### 2.1. Subjects and Clinical Examinations

An 18-year-old girl (proband 1 from family 1) attended Shenzhen Eye Hospital because of severe myopia and blurred vision. In addition, a 9-year-old boy (proband 2 from family 2) attended Shenzhen Eye Hospital because of headache and ophthalmodynia without obvious causes. Slit lamp examination, vision and intraocular pressure testing, and general physical check-up were performed in proband and her/his parents. Blood Hcy level was measured by biochemical assays. Quantitative analysis of homocysteine metabolites and associated coenzyme factors was conducted using the AB Sciex Triple Quad^TM^ 4500 LC-MS/MS system (Shanghai AB SCIEX Analytical Instrument Trading Co., Shanghai, China) following the manufacturer’s operational protocol. This study was approved by the Shenzhen Eye Hospital Ethics Committee (Approval Code: 2021110402-02) and was conducted in accordance with the Declaration of Helsinki. Written informed consent was obtained from all patients and controls.

### 2.2. Whole Exome Sequencing (WES)

Genomic DNA (gDNA) samples were extracted from peripheral blood samples of 8 Chinese individuals (i.e., proband 1 and her 3 family members from family 1, and proband 2 and his 3 family members from family 2, respectively) by using MagPure Buffy Coat DNA Midi KF Kit (Magen, Guangzhou, China) and assessed by a NanoDrop spectrophotometer (Thermo Fisher Scientific, Waltham, MA, USA). Libraries were prepared using the MGIEasy DNA Library Prep Kit (BGI-Shenzhen, China). Exome capture was performed using the KAPA HyperExome V2 Probes (Roche, Basel, Switzerland) and sequenced on a MGISEQ-2000 platform (BGI-Shenzhen, China). After sequencing, the clean next generation sequencing (NGS) data were aligned with the human reference genome (GRCh37/hg19) genome using Burrows–Wheeler alignment (BWA) program (version 0.7.17). Single nucleotide variants (SNV) and insertion/deletion (Indel) calls, as well as genotypes detection were conducted using the GATK tool (version 4.0). Finally, the mutations in the *CBS* gene carried by these families were further confirmed by Sanger sequencing.

### 2.3. Bioinformatics Analysis

The conservation of a mutation site was analyzed using the National Center for Biotechnology Information (NCBI) BLAST protein blast tool. Multiple sequence alignment from 8 species was performed using the Geneious Prime (version 2022.2.1). To assess the presumptive effect of this variant on splicing, the following three different in silico prediction tools were used: Human Splicing Finder-Version 3.1 (HSF, http://www.umd.be/HSF3/HSF.shtml; accessed on 23 May 2024), Varseak (https://varseak.bio/index.php; accessed on 23 May 2024), and SpliceAI (https://spliceailookup.broadinstitute.org/; accessed on 23 May 2024).

### 2.4. Minigene Assays

Wild-type and mutant minigene plasmids were constructed for the *CBS* mutation (c.1359-1G>C) using two different vectors (pcMINI and pcDNA3.1). The target mutation was introduced by site-directed mutagenesis using a PrimeStar mutagenesis basal kit (TaKaRa, Otsu, Japan), according to the manufacturer’s instructions. The sequences of exon 14, intron 14, exon 15, and part of intron 15 were amplified and inserted into the pcMINI vector to generate the minigene constructs: pcMINI-CBS-wt/mut. In addition, the sequences of exon 14, intron 14, exon 15, intron 15, and exon 16 were amplified and inserted into the pcDNA3.1 vector to generate the minigene constructs: pcDNA3.1-CBS-wt/mut. The sequences of all the recombinant vectors were confirmed by Sanger sequencing. Then, the four plasmids were transfected into MCF-7 and HEK 293T cell lines using Lipofectamine 3000 (Invitrogen, Carlsbad, CA, USA). Total RNA was isolated using the Trizol reagent (Invitrogen, USA) from the cells transfected for 48 h and reverse transcription was performed using Hifair^®^ II 1st Strand cDNA Synthesis SuperMix (gDNA digester plus) (Yeasen, Shanghai, China). The cDNA was amplified by PCR, and PCR products were analyzed by electrophoresis on a 1.5% agarose gel and Sanger sequencing. All primers used in the minigene splicing assay are listed in Supplementary Table S1.

### 2.5. Protein Structural Modeling

To predict the structural changes in the identified mutation, we obtained structural models of the wild-type and two mutant CBS proteins using the AlphaFold software (version 2.1). The predicted protein structures were visualized via PyMOL 3.1 software (https://www.pymol.org/).

### 2.6. Measurement of Homocysteine (Hcy) and CBS Enzyme Levels

Whole blood samples collected in EDTA anticoagulation tubes were centrifuged at 3000× *g* for 15 min to obtain plasma samples. The Hcy levels and CBS enzyme activities were assessed in the plasma of all 8 participants and 5 normal controls using the Hcy enzyme-linked immunosorbent assay (ELISA) kit and CBS ELISA kit according to manufacturers’ instructions, respectively (My BioSource, San Diego, CA, USA). Samples with Hcy levels or CBS enzyme activity above the upper limit of the measurement range were diluted with normal saline and quantified again. The Optical Density (O.D.) at 450 nm was read using an Multiskan FC Microplate Reader (Thermo Scientific, Waltham, MA, USA).

### 2.7. Construction of CBS Expression Plasmids

The wild-type Flag-CBS expression plasmid was generated by fusing the full-length wild-type CBS cDNA fragment into pcDNA3.1 plasmid (Bioeagle, Wuhan, China) with an N-terminal 3×Flag tag. Five variants (c.1358_1359ins154bp, c.1359_1467del, c.833T>C, c.131delT, c.919G>A) were introduced into the wild-type cDNA using site-directed mutagenesis, respectively. Then, the mutant CBS cDNA was cloned into pcDNA3.1 plasmid (Bioeagle, Wuhan, China) with an N-terminal 3×Flag tag to construct mutant Flag-CBS expression plasmid. The sequences of all plasmids were confirmed by Sanger sequencing.

### 2.8. Cell Culture and Transfection

HEK293T cells were cultured in Dulbecco’s Modified Eagle Medium (DMEM; Gibco, Thermo Fisher Scientific, USA) with 10% fetal bovine serum (FBS) and 1% penicillin–streptomycin at 37 °C and 5% CO_2_. After the cells were 50–70% confluent, they were transiently transfected with plasmid (containing either wild-type or two mutant CBS) using Lipofectamine 3000 reagent (Invitrogen, USA) according to manufacturer’s protocols. Approximately 24 h after transfection, the cells were collected for subsequent experiments.

### 2.9. RNA Extraction and Quantitative Reverse Transcription-Polymerase Chain Reaction (PCR)

Total RNA was extracted from peripheral leukocytes of the probands, their family members, or the unrelated healthy individuals, as well as from the collected HEK293T cells using Trizol (Takara, Kusatsu, Japan). The cDNA was synthesized using the Hifair^®^ II 1st Strand cDNA Synthesis SuperMix kit (gDNA digester plus) (Yeasen, Shanghai, China) according to the manufacturer’s protocol. qRT-PCR was performed using Hieff^®^ qPCR SYBR Green Master Mix (Yeasen, Shanghai, China) on the Step-OnePlusTM Real-time PCR System (Applied Biosystems, Foster City, CA, USA). The reaction conditions were as follows: 5 min at 95 °C, followed by 40 cycles consisting of 10 s at 95 °C and 30 s at 60 °C. Each qRT-PCR assay was performed in triplicate, and the glyceraldehyde-3-phosphate dehydrogenase (GAPDH) gene was used as the normalization control. All primers used in the qRT-PCR are shown in Supplementary Table S2. The relative gene expression level was calculated using the 2^−ΔΔCT^ method. The significance was determined using a *t*-test, with *p* < 0.05 considered statistically significant. The primers used are listed in Supplementary Table S2.

### 2.10. Western Blot

Total protein was extracted using RIPA lysis buffer (Beyotime, Nantong, China) mixed with 1% protease inhibitor and phosphorylase inhibitor on ice, and concentrations of protein were determined using a BCA Kit (Thermo Fisher Scientific, Shanghai, China). A measure of 20 μg total protein was separated by 10% SDS-PAGE and transferred to the PVDF membrane and then blocked for 1 h (5% BSA) at room temperature. The membrane was incubated with the Flag Tag Mouse Monoclonal antibody (1:2500; #66002; Proteintech, Chicago, IL, USA) and GAPDH (1:5000, 10494-1-AP, proteintech, Chicago, IL, USA) primary antibodies at 4 °C overnight. After washing with TBST and incubation with secondary antibodies (1:5000, 7074s, CST), the membrane was covered with ECL reagents (Thermo Fisher Scientific, Shanghai, China) and visualized using the GeneGnome XRQ Chemical Imaging System (Gene Company Limited, Hong Kong, China). The relative intensity was analyzed using ImageJ software version 1.8.0 (BioRad, Berkeley, CA, USA) and standardized with GAPDH. The statistical significance of the data was determined using a *t*-test, *p* < 0.05 was considered statistically significant.

### 2.11. Statistical Analysis

Statistical analysis was performed with GraphPad Prism 9 (GraphPad Software, Inc., San Diego, CA, USA). Data represent at least three independent experiments. Graphs are presented as mean values ± standard deviation (SD) (error bars) and individual values (dots) (scatter plot with error bars). Statistical significance was determined using Student’s *t*-test or one-way analysis of variance (ANOVA), followed by Tukey’s post hoc tests. *p* < 0.05 was considered statistically significant.

## 3. Results

### 3.1. Clinical Presentations

**Figure 1:**
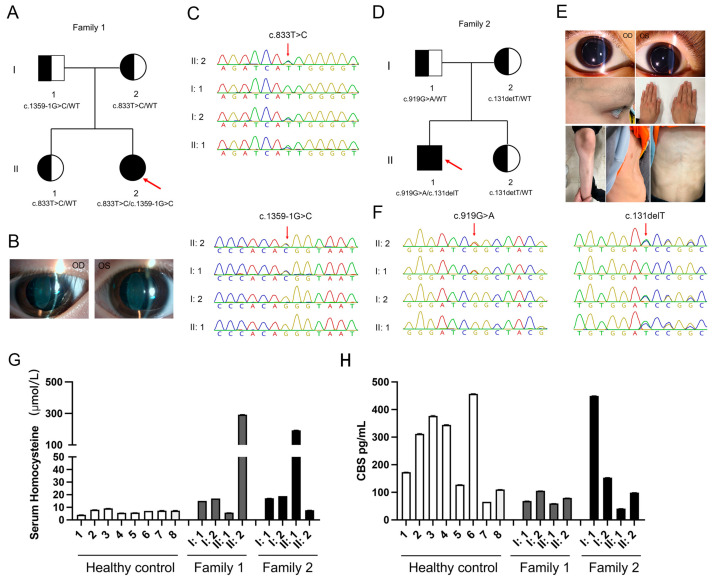
Pedigree of the family and the clinical features of the proband. (**A**) The pedigree of the family 1. The proband (II: 2) is marked with a red arrow. (**B**) An anterior segment photograph of the proband (II: 2) in family 1 illustrated ectopia lentis. (**C**) Sanger sequencing chromatogram of the CBS mutations from the proband in family 1 and her parents and sister. The red arrow represents the location of the mutation site. (**D**) The pedigree of family 2. The proband (II: 1) is marked with a red arrow. (**E**) Clinical characteristics of the proband (II: 1) in family 2. (**F**) Sanger sequencing chromatogram of the CBS mutations from the proband in family 2 and his parents and sister. The red arrow represents the location of the mutation site. (**G**) Plasma homocysteine levels quantified by ELISA showing abnormal increase in two probands. (**H**) Plasma CBS enzyme levels quantified by ELISA showing a decrease in two probands compared to the average control level.

The proband 1 (Family 1, II: 2) was brought by her parents with severe myopia and decreased vision in both eyes. There was no history of trauma or similar family history. Her slit lamp examination and vision testing showed lens dislocation and high-grade myopia (−19.0 D) and her best-corrected visual acuity was 0.4 (Figure 1A,B). Her intraocular pressure (IOP) was normal. Other systemic symptoms included osteoporosis and tremor of both hands for nearly a decade, as well as liver and thyroid cysts. Her plasma Hcy levels were 306.26 μmol/L (reference range < 15 μmol/L). The levels of vitamin B6 and 5-methyltetrahydrofolate in plasma of two probands were both lower than normal; methionine in the plasma of two probands were both higher than normal (Table 1).

The proband 2 (a 9-year-old boy, Family 2, II: 1) was urgently admitted to Shenzhen Eye Hospital because of headache and ophthalmodynia without obvious causes. Slit lamp examination and vision testing showed he has lens dislocation and high-grade myopia (−24.0 D) and his best-corrected visual acuity was 0.12 (Figure 1D,E). IOP testing showed that the IOP of his right and left eyes are 13 mm Hg and 47 mm Hg. We immediately performed IOP reduction on the patient, followed by timely phacoemulsification and intraocular lens replacement surgery. Physical examination of proband reveals that intellectual development retardation, spider-like fingers, slender bones, severe pectus excavatum, mild scoliosis, skin reticular bluish spots, slightly curly and fine hair. The parents and sister of the proband were unaffected and there was no family history.

As shown in Figure 1G,H, the total plasma Hcy levels (μmol/L) and CBS levels (pg/mL) of all participants are indicated. Two probands and some family members exceeded the normal plasma cysteine level (reference range < 15 μmol/L) and the two probands and their family members all exhibited CBS levels that were lower than normal (mean ± standard error: 245.89 ± 141.45 pg/mL). Other physical examination indicated that the family members of the probands were unaffected. After diagnosis, the two affected probands have implemented strict dietary regimens comprising a low-protein diet with supplementation of vitamin B6-enriched foods, accompanied by pharmacotherapy including high-dose vitamin B6 (300 mg three times a day), betaine (2 g twice a day), and folic acid (5 mg twice a day) to mitigate hyperhomocysteinemia. At the time this report was submitted, their physical condition was stable.

### 3.2. Bioinformatics Analysis of Functional Effects of Genetic Variants

**Figure 2:**
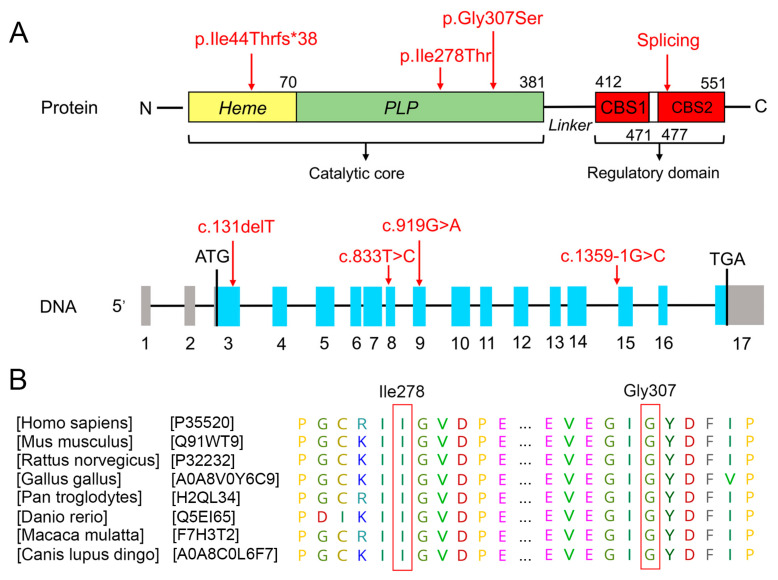
Identification and analysis of mutations in the *CBS* gene. (**A**) Location of four *CBS* mutations c.833T>C, c.1359-1G>C, c.919G>A, and c.131delT at the nucleotide and protein levels (the protein and DNA structures are not drawn in scale) [12]. (**B**) Multiple species sequence alignments of CBS proteins by the Geneious Prime (version 2022.2.1) showed the high conservations of residue 278 (Ile) and residue 307 (Gly), respectively. The CBS protein UniportKB IDs (Uniport database URL: https://www.uniprot.org/) for eight species are displayed.

Through WES, one compound heterozygous CBS variant [c.833T>C (p.Ile278Thr) and c.1359-1G>C] was identified in the first proband (Family 1, II: 2), and another compound heterozygous CBS variant [c.919G>A(p.Gly307Ser and c.131delT(p.Ile44Thrfs*38)] was identified in the second proband (Family 2, II: 1). Sanger sequencing confirmed this result, showing that the c.833T>C and c.131delT variants were inherited from their respective mothers (Figure 1C), while the c.1359-1G>C and c.919G>A variants were inherited from their respective fathers (Figure 1F). The *CBS* gene consists of 15 coding exons, and codes for a 551 amino acid protein compose of a catalytic core including the heme and pyridoxal 5′-phosphate (PLP) cofactors, and a regulatory domain including CBS-1 and CBS-2 domain (UniProtKB-P35520.1, Figure 2A). The two domains are connected by a 32-residue long interconnecting linker (Figure 2A). The c.131delT, c.833T>C, c.919G>A, and c.1359-1G>C variants are located in the coding sequence at exon 3, 8, and 9 and intron 14 of CBS, respectively (Figure 2A). The c.833T>C (p.Ile278Thr) and c.919G>A(p.Gly307Ser) were located in the catalytic core structural domain of the CBS protein (Figure 2A). The c.131delT (p.Ile44Thrfs*38) was predicted to not affect *CBS* RNA splicing and c.1359-1G>C was predicted to generate aberrant splicing transcripts of *CBS* gene (Table 2). A comparison of the amino acid sequences indicates that the 278th amino acids (Isoleucine) and 307th amino acids (Glycine) are highly evolutionarily conserved among different species (Figure 2B).

### 3.3. Minigene Assay

**Figure 3:**
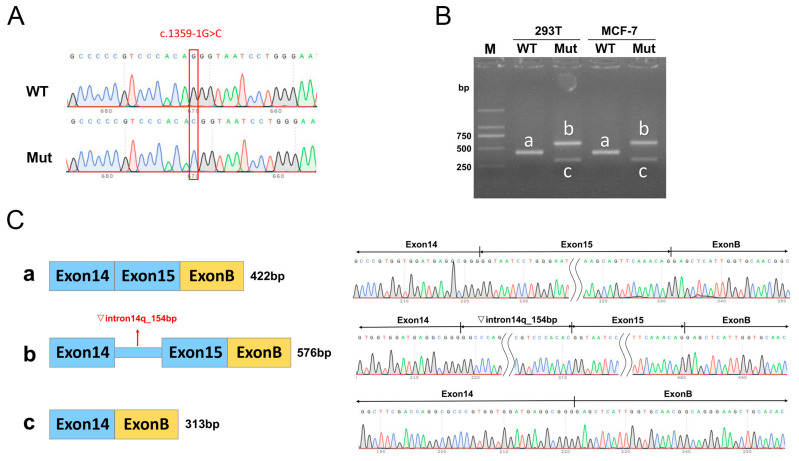
Minigene assay and Sanger sequencing of spliced transcripts based on pcMINI-N-CBS-wt/mut recombinant vectors. (**A**) Sanger sequencing results of the minigene constructs. (**B**) RT-PCR products of the minigenes expressed in 293T and MCF-7 cells. (**C**) Minigene splicing diagram and Sanger sequencing of the amplified products. Exon B is a component of the pcMINI-N vector.

**Figure 4:**
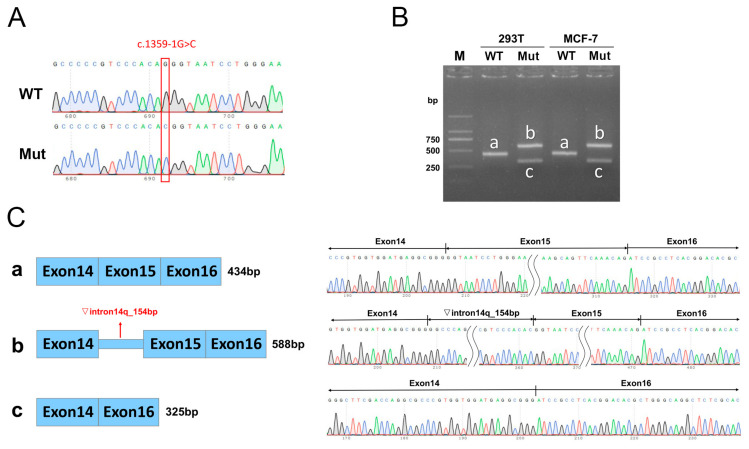
Minigene assay and Sanger sequencing of spliced transcripts based on pcDNA3.1-CBS-wt/mut recombinant vectors. (**A**) Sanger sequencing results of the minigene constructs. (**B**) RT-PCR products of the minigenes expressed in 293T and MCF-7 cells. (**C**) Minigene splicing diagram and Sanger sequencing of the amplified products.

The wild-type and mutant minigenes were inserted into the pcMINI-N and pcDNA3.1 vectors, respectively, and the successfully constructed recombinant vectors were validated by Sanger sequencing (Figure 3A and Figure 4A). The recombinant vector was transfected into MCF-7 and HEK-293T cell lines, and samples were collected 48 h after transfection for RNA extraction and amplification. The RT-PCR amplification products from the MCF-7 and HEK-293T cultures transfected with the vectors carrying the mutation showed two distinct bands: one larger than the wild-type band size (labeled as band b) and the other smaller than the wild-type band size (labeled as band c, Figure 3B and Figure 4B). All obtained bands were subjected to Sanger sequencing, showing that the wild-type band a was normal splicing, with the splicing pattern of Exon 14 (135 bp)-Exon 15 (109 bp)-Exon B (57 bp), while the mutant band b showed a 154 bp retention on the right side of intron 14, with the splicing pattern of Exon 14 (135 bp)-▽intron 14 (154 bp)-Exon 15 (109 bp)-Exon B (57 bp) (Figure 3C and Figure 4C). The mutant band c showed Exon 15 skipping, with the splicing pattern of Exon 14 (135 bp)-Exon B (57 bp) (Figure 3C and Figure 4C).

### 3.4. Protein Structure Analysis

**Figure 5:**
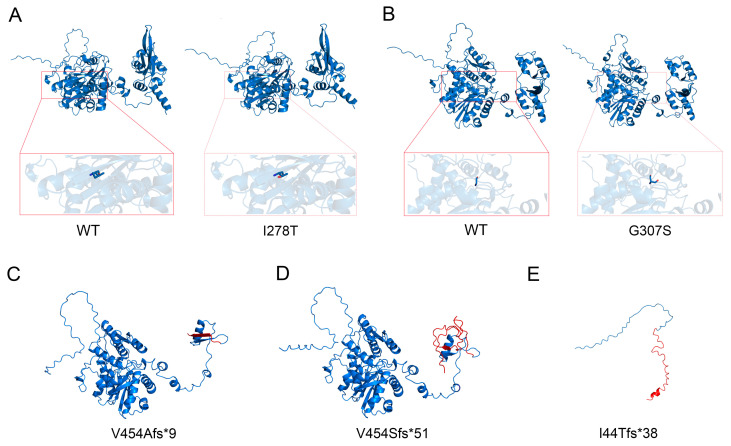
Structural models for wild-type and mutant CBS proteins were constructed using AlphaFold software (version 2.1) and visualized using PyMOL software (version 3.1, https://www.pymol.org/). (**A**) Structural models for wild-type and CBS proteins with I278T mutation. (**B**) Structural models for wild-type and CBS proteins with G307S mutations. (**C**) Structural models for CBS protein with V454Afs*9 mutation. (**D**) Structural model for CBS protein with V454Afs*51 mutation. (**E**) Structural model for CBS protein with I44Tfs*38 mutation. The amino acids located before and after the mutation are indicated in blue and red, respectively.

The c.1359-1G>C splicing variation in CBS generates two truncated proteins, including the mutant type 1 (Mut-1) which resulted in the premature termination codon p.Val454Alafs*9, which results in a truncated protein with a length of 461 amino acids, while the mutant type 2 (Mut-2) resulted in the premature termination codon p.Val454Serfs*51 of CBS protein, which results in a truncated protein with a length of 503 amino acids. Using protein structural modeling analysis, we compared the structural differences in the wild-type CBS protein, the two above-mentioned truncated proteins, CBS mutation protein with Ile278Thr, CBS mutation protein with p.Gly307Ser, and CBS mutation protein with Ile44Thrfs*38 (Figure 5).

### 3.5. In Vitro Expression Analysis

**Figure 6:**
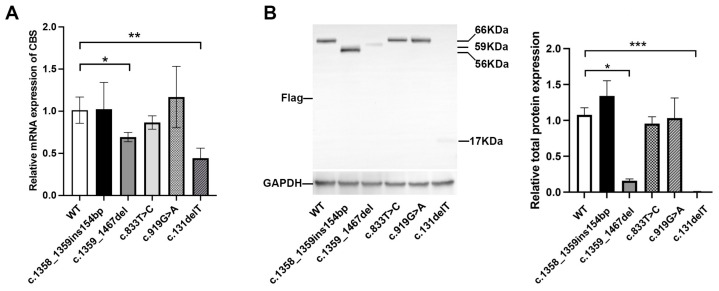
In vitro expression experiments of wild-type and mutant *CBS* in HEK-293T cells. (**A**) Detection of *CBS* mRNA expression of wild-type versus two mutant CBS by qRT-PCR analysis. (**B**) Detection of protein expressions of wild-type versus two mutant CBS by Western blotting. * represents *p* < 0.05, ** represents *p* < 0.01, *** represents *p* < 0.001.

To evaluate the impact of the variants identified in this study on CBS expression, we compared the mRNA and protein levels of the wild-type and mutant CBS using expression plasmids transfected into HEK-293T cells. Our results showed that the c.1359_1467del and c.131delT variants both caused a significant decrease (*p* < 0.05) in both mRNA and protein expression compared to the wild type, while other three variants (c.1358_1359ins154bp, c.833T>C, and c.919G>A) had no significant effect on CBS expression in vitro (Figure 6A,B).

## 4. Discussion

Classic homocystinuria, a rare congenital metabolic disorder caused by cystathionine beta synthase (CBS) enzyme deficiency, was associated with high blood Hcy concentration, thromboembolic tendency, and neurocognitive symptoms [5]. Hcy is an intracellular intermediate product that is not normally detected in plasma or urine. However, when the conversion of Hcy to methionine or the conversion process of cysteine are blocked, it accumulates outside the cell and leads to homocystinuria [13]. Two probands in this study both presented with spherical lenses and their plasma Hcy levels were both higher than the normal level. According to reports, approximately 40% of 5-year-old untreated homocystinuria patients have lens subluxation, and almost all patients exhibit it before the age of 25 [14]. Spherical lens is a disease of the suspensory ligament lens, which can cause severe high myopia and easily lead to lens dislocation. The suspensory ligament of the lens is mainly composed of fibrillar protein 1, which contains approximately 13% cysteine residues. The elevated Hcy level leads to a reduction in the C-terminal of fibrillin-1, causing abnormal self-interaction, ultimately resulting in abnormal function of the suspensory ligament [15].

The human *CBS* gene, located at chromosome 21q22.3, encodes a 551-amino-acid CBS protein and participates in the transsulfuration pathway responsible for diverting Hcy to the biosynthesis of cysteine and generating H_2_S [16]. The CBS enzyme consists of a catalytic core that includes the heme and PLP cofactors, and a regulatory domain that includes CBS-1 and CBS-2 [17,18,19]. A large number of missense mutations were identified in the catalytic core of CBS [20]. Several C-terminal regulatory domain variants did not reduce the catalytic activity but were insensitive to further activation of S-adenosylmethionine (AdoMet), which binds to CBS C-terminal regulatory domain [21]. The connecting linkers between the N- and the C-terminal domains are important in stabilizing the higher-order oligomeric structure of CBS and enabling AdoMet-dependent regulation [22].

The first mutation in the human *CBS* gene was reported by Kozich and Kraus in 1992 [23]. Most affected patients have compound heterozygous mutations in CBS [24,25,26,27]. To date, very few mutations from Chinese cases with classical homocystinuria have been reported [25,27,28,29]. In this study, four mutations—c.833T>C (p.Ile278Thr), c.1359-1G>C, c.131delT (I44Tfs*38), and c.919G>A (p.Gly307Ser)—were identified in two Chinese probands with classic homocystinuria. The c.1359-1G>C and c.131delT variations have not been reported, and the c.833T>C and c.919G>A variations were previously reported and constitute two of the most prevalent mutations in European patients [30,31,32]. Patients with c.833T>C mutation only exhibit lens ectopia and mild bone demineralization, which is related to the retention of some degree of pyridoxine reactivity in the patient [30]. Homozygous individuals with c.919G>A mutations often have severe defects and are non-responsive to pyridoxine [33]. Interestingly, there is currently no research report on the detection of c.833T>C and c.919G>A mutations in Chinese patients with classic homocystinuria, although one study reported that the c.833T>C mutation was present in a Chinese patient from Hong Kong with homocystinuria responsive to pyridoxine [28]. The frequency differences in these mutations in the Chinese population suggest that there may be racial differences in the distribution of CBS gene mutations, and it is therefore necessary to screen for mutations in a larger group of patients with homocystinuria.

Based on software prediction, c.1359-1G>C affects CBS splicing, while c.131delT prediction does not affect CBS splicing. The minigene experiments revealed that c.1359-1G>C mutation resulted in retaining a 154 bp DNA fragment (c.1358_1359ins154bp) on the right side of intron 14 or in the skipping of exon 15 (c.1359_1467del). In terms of protein structure, both mutations led to changes in the reading frame, generating premature termination codons (PTCs), forming two truncated proteins p.Val454Alafs*9 and p.Val454Serfs*51. In addition, we investigated the effects of overexpression of five different CBS mutations on CBS expression levels in HEK293T cells. The results showed that c.1359_1467del and c.131delT mutations significantly reduced CBS mRNA and protein levels, while the other three mutations did not cause significant changes.

The two probands in this study exhibited different clinical phenotypes, which were directly related to the different CBS mutations they carried. In plasma, the total homocysteine (tHcy) levels in Tg-G307S CBS ^−/−^ and Tg-I278T CBS ^−/−^ mice were similar to those in control animals, but the elevated methionine levels were increased in Tg-G307S CBS ^−/−^ mice [34]. Recombinant protein p.Gly307Ser-CBS has been synthesized using several different heterologous expression systems and in almost all cases, heterologous proteins lack enzymatic activity. In an experiment using native gel, p.Gly307Ser effectively forms polymer. Therefore, Gly307Ser substitution may impair catalytic function by limiting the ability of tyrosine at position 308, rather than causing folding defects [33].

## 5. Conclusions

Overall, we revealed the clinical manifestations of two patients with homocystinuria and conclude that the compound heterozygous mutation of CBS gene is the cause of the disease. Through in vitro minigene experiments, we analyzed the effects of splice mutations on the level of CBS transcripts and protein function and further confirmed the role of CBS in the pathogenesis of homocystinuria. This study has extended the mutation spectrum of CBS induced homocysteinuria and has potential implications for developing new diagnostic and therapeutic methods.

## Figures and Tables

**Table 1:** Plasma levels of homocysteine metabolites and coenzyme in two probands.

Plasma Measure	Proband 1	Proband 2	Reference Range	Unit
Vitamin B2	3.35	2.97	2.3–14.6	ng/mL
Vitamin B6	1.85	1.03	3–30	ng/mL
Vitamin B9	3.97	4.31	>4	ng/mL
Vitamin B12	51.49	24.58	<52.91	ng/mL
5-methyltetrahydrofolate	3.7	2.46	>4	ng/mL
Methionine	95.69	73.35	4–44	μmol/L
Cystathionine	0.16	1.27	<5	μmol/L

**Table 2:** In silico analysis of the *CBS* variants c.1359-1G>C and c.131delT.

Splicing prediction		c.1359-1G>C		c.131delT	
	Software	ΔScore	Effect	ΔScore	Effect
	HSF (Version 3.1)	/	+	/	-
	Varseak	/	+	/	-
	SpliceAI	0.98	+	0.00	-

## Data Availability

The original contributions presented in the study are included in the article/Supplementary Materials, further inquiries can be directed to the corresponding authors.

## Supplementary Materials

The following supporting information can be downloaded at: https://www.mdpi.com/article/10.3390/biomedicines13040919/s1, Table S1. Primer sequences used to amplify *CBS* genomic fragments; Table S2. Primer sequences used for qRT-PCR.
