# Supplementary material for: Identification and Functional Analysis of Cystathionine Beta-Synthase Gene Mutations in Chinese Families with Classical Homocystinuria
Source: Biomedicines. 2025 Apr 9;13(4):919. doi: 10.3390/biomedicines13040919 (PMC12024673; doi:10.3390/biomedicines13040919)
Supplement: Supplementary file 1 [file biomedicines-13-00919-s001.zip › Supplementary Table S1.pdf]

Supplementary Table S1. Primer sequences used to amplify *CBS* genomic fragments

Process	Name of Prime	Primer sequence (5'→3')
Step 1 of the NEST-PCR	12692-CBS-F	TGCTCACACACGCTTGTTGG
	12958-CBS-F	AAGAAGCCCTGGTAAGACCG
Step 2 of the NEST-PCR	15652-CBS-R	CTGAGCCTCTGCATGCTGGA
	15868-CBS-R	GATGCGCTCAGGAGTTGCAA
Amplify the pcMINI-CBS-wt	pcMINI-N-CBS-BamHI-F	GCTCGGATCCATGTGGTGGTGGCACCTCCGTGTTC
	pcMINI-N-CBS-EcoRI-R	TGCAGAATTCCCGCCTTCGAGGCTGGAGTA
Amplify the left half of pcMINI mutant	pcMINI-N-CBS-BamHI-F	GCTCGGATCCATGTGGTGGTGGCACCTCCGTGTTC
	CBS-mut-R	ATTCCCAGGATTACCGTGTGGGACGGGGGCA
Amplify the right half of pcMINI mutant	CBS-mut-F	TGCCCCCGTCCCACACGGTAATCCTGGGAAT
	pcMINI-N-CBS-EcoRI-R	TGCAGAATTCCCGCCTTCGAGGCTGGAGTA
Amplify the pcDNA3.1-CBS-wt	pcDNA3.1-CBS-HindIII-F	ACTTAAGCTTATGTGGTGGTGGCACCTCCGTGTTC
	pcDNA3.1-CBS-XhoI-R	TAGACTCGAGGTACTGGATCTGCTCGTGCACC
Amplify the left half of pcDNA3.1 mutant	pcDNA3.1-CBS-HindIII-F	ACTTAAGCTTATGTGGTGGTGGCACCTCCGTGTTC
	CBS-mut-R	ATTCCCAGGATTACCGTGTGGGACGGGGGCA
Amplify the right half of pcDNA3.1 mutant	CBS-mut-F	TGCCCCCGTCCCACACGGTAATCCTGGGAAT
	pcDNA3.1-CBS-XhoI-R	TAGACTCGAGGTACTGGATCTGCTCGTGCACC
PCR amplifications of pcMINI-N vector	pcMINI-N-F	CTAGAGAACCCACTGCTTAC
	pcMINI-N-R	GCCCTCTAGACTGGTCATTCCGGCTC
PCR amplifications of pcDNA3.1 vector	pcDNA3.1-F	CTAGAGAACCCACTGCTTAC
	pcDNA3.1-R	TAGACTCGAGGTACTGGATCTGCTCGTGCACC
