# Supplementary material for: Identification and Functional Analysis of Cystathionine Beta-Synthase Gene Mutations in Chinese Families with Classical Homocystinuria
Source: Biomedicines. 2025 Apr 9;13(4):919. doi: 10.3390/biomedicines13040919 (PMC12024673; doi:10.3390/biomedicines13040919)
Supplement: Supplementary file 1 [file biomedicines-13-00919-s001.zip › Supplementary Table S2.pdf]

Supplementary Table S2. Primer sequences used for qRT-PCR.

**Name of Prime**	**Primer sequence (5'→3')**	Product length (bp)
CBS-F	AAGGGGAGCCTGGAGAAGG	125
CBS-R	TGATGTGGGGACTCGGAGG	
GAPDH-F	TGTGGGCATCAATGGATTTGG	116
GAPDH-R	ACACCATGTATTCCGGGTCAAT	
